# Optimal cut-off criteria for duplex ultrasound for the diagnosis of restenosis in stented carotid arteries: *Review and protocol for a diagnostic study*

**DOI:** 10.1186/1471-2377-9-36

**Published:** 2009-07-22

**Authors:** Paul J Nederkoorn, Martin M Brown

**Affiliations:** 1 Department of Neurology, Academic Medical Center, Amsterdam, The Netherlands; 2 UCL Institute of Neurology, the National Hospital for Neurology and Neurosurgery, London, UK

## Abstract

**Background:**

Carotid angioplasty with stenting is a relatively new, increasingly used, less-invasive treatment for the treatment of symptomatic carotid artery stenosis. It is being evaluated in ongoing and nearly finished randomized trials. An important factor in the evaluation of stents is the occurrence of in-stent restenosis. An un-stented carotid artery is likely to have a more elastic vessel wall than a stented one, even if stenosis is present. Therefore, duplex ultrasound cut-off criteria for the degrees of an in-stent stenosis, based on blood velocity parameters, are probably different from the established cut-offs used for un-stented arteries. Routine criteria can not be applied to stented arteries but new criteria need to be established for this particular purpose.

**Methods/Design:**

Current literature was systematically reviewed. From the selected studies, the following data were extracted: publication year, population size, whether the study was prospective, duplex ultrasound cut-off criteria reported, which reference test was used, and if there was an indication for selection bias and for verification bias in particular. Previous studies often were retrospective, or the reference test (DSA or CTA) was carried out only when a patient was suspected of having restenosis at DUS, which may result in verification bias.

**Results:**

In general, the velocity cut-off values for stenosis measurements  in stented arteries were higher than those reported for unstented arteries. Previous studies often were retrospective, or the reference test (DSA or CTA) was carried out only when a patient was suspected of having restenosis at DUS, which may result in verification bias.

**Discussion:**

To address the deficiencies of the existing studies, we propose a prospective cohort study nested within the International Carotid Stenting Study (ICSS), an international multi-centre trial in which over 1,700 patients have been randomised between stenting and CEA. In this cohort we will enrol a minimum of 300 patients treated with a stent. All patients undergo regular DUS examination at the yearly follow-up visit according to the ICSS protocol. To avoid verification bias, an additional computed tomography angiography (CTA) will be performed as a reference test in *all *consecutive patients, regardless of the degree of stenosis on the initial DUS test.

## Background

Carotid endarterectomy (CEA) is an effective and established treatment for secondary prevention of stroke in patients with symptomatic carotid artery stenosis[1]. Carotid angioplasty with stenting (CAS) is a relatively new, increasingly used, less-invasive treatment, which is being evaluated in ongoing or nearly finished randomized trials, such as the Carotid Revascularization Endarterectomy versus Stent Trial (CREST) and the International Carotid Stenting Study (ICSS) [2,3]. ICSS finished randomization and will publish its safety results in the short term. Previous trials that compared CEA with CAS were rather heterogeneous and not large enough to allow reliable conclusions. Furthermore, because there is limited follow-up information to date, the long-term effect of CAS remains unclear. We therefore need more data from these nearly finished randomised trials, including long follow-up, before recommending if and when stenting should replace endarterectomy in clinical practice [4,5].

An important factor in the evaluation of stents is the occurrence of in-stent restenosis. Both the Carotid and Vertebral Artery Transluminal Angioplasty Study (CAVATAS) and the Stent-Protected Angioplasty versus Carotid Endarterectomy (SPACE) study reported a higher incidence of restenosis in patients treated with CAS than in patients treated with CEA [6,7]. However, we do not know if an in-stent restenosis will give rise to symptoms in a similar way to atherosclerotic carotid stenosis, and the clinical consequences of restenosis are yet unknown. In CAVATAS the majority of patients in the endovascular arm were treated by angioplasty without stenting. The SPACE-investigators concluded that it could not be excluded that the degree of in-stent stenosis was slightly overestimated by conventional ultrasound criteria. Long term follow-up data of patients treated with a stent are needed to investigate the clinical consequences of in-stent restenosis. Prior to these analyses, to reliably diagnose in-stent restenosis, we first need reliable duplex ultrasound cut-off criteria.

Traditionally, the degree of stenosis in an untreated carotid artery was measured with conventional digital subtraction angiography (DSA). Because of a small but non-negligible risk of stroke or death, DSA has been replaced by non-invasive tests, such as duplex ultrasound (DUS), CT angiography (CTA) or MR angiography (MRA) [8-10]. In the follow-up of patients with a stent, DUS is often used to monitor the patency of the stent and the occurrence of in-stent restenosis. For routine evaluation of un-stented carotid arteries, DUS is a well validated diagnostic test and the cut-off criteria for the different degrees of stenosis are clear [8,9]. For measurements within stents, however, these criteria may not suffice.

**Figure 1:**
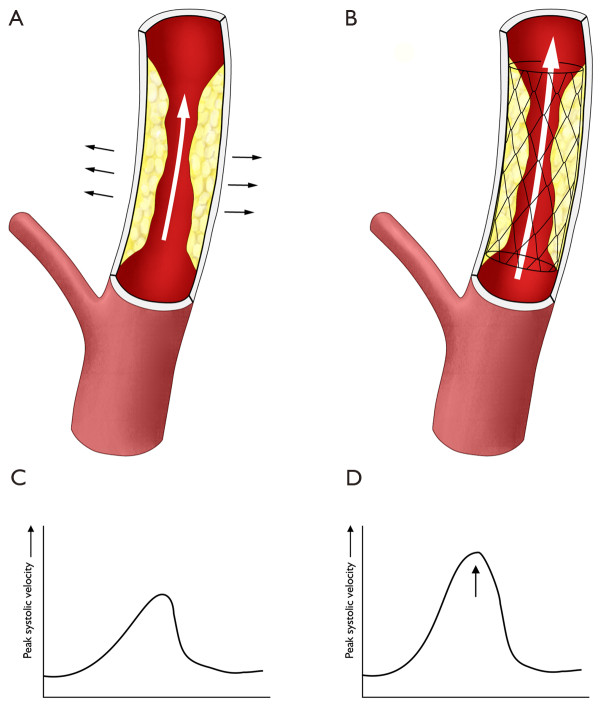
**Illustration of our hypothesis that a un-stented carotid artery (A) has a more elastic vessel wall than a stented one (B), and that the PSV raises more in a stented (D) than in an un-stented carotid artery (C) with a similar degree of stenosis**.

In a stenosed artery, narrowing of the lumen results in higher blood flow velocities at that point. Estimating the degree of stenosis with DUS is based on this principle. The peak systolic velocity (PSV) is the best predictor for the severity of the stenosis [11]. However, the degree of restenosis in a stented carotid artery, measured according to the North American Symptomatic Carotid Endarterectomy Trial (NASCET) criteria on CTA or MRA, is often less severe than expected based on the PSV in the DUS test. Possibly, blood flow and blood turbulence behave differently in an artificial stent than in a normal vessel. This problem has been addressed in literature before; particularly by Lal en coworkers [12]. An un-stented carotid artery is likely to have a more elastic vessel wall than a stented one, even if stenosis is present. The cut-off criteria for the degrees of an in-stent stenosis, based on blood velocity parameters, are probably different from the established cut-offs used for un-stented arteries. We hypothesise that the PSV raises more in a stented than in a un-stented carotid artery with a similar degree of stenosis (figure 1). Additional duplex characteristics may also be different in an in-stent restenosis, such as formation of intimal hyperplasia instead of atherosclerotic plaque as cause of the stenosis. These characteristics need to be studied as well; the present study, however, will be limited to definition of PSV criteria. Routine criteria can not be applied to stented arteries but new criteria need to be established for this particular purpose.

Valid criteria are needed for future research and clinical decisions in patients treated with a carotid artery stent. In this article, we therefore first review the current literature and discuss the most important limitation of earlier diagnostic studies on this topic, verification bias. In the discussion, we describe the design of a new diagnostic study designed to validate the use of DUS in-stent stenosis measurements during follow-up after CAS and to determine reliable cut-off criteria for the different degrees of stenosis.

## Methods/Design

The PubMed databases have been searched from 2000 until 2009 for publications with "duplex ultrasound" combined with "carotid", "stent" or "in-stent", and "restenosis" as keywords, without language restrictions. Cross-references and review articles were used for search completion. In case of more than one publication on this topic by one group, the most recent or largest series was chosen. A hand-search of relevant journals and conference proceedings was not performed. Based on titles and abstracts, studies evaluating duplex ultrasound for assessment of in-sent restenosis were selected. To be included in this review, the study needed to provide duplex ultrasound cut-off criteria, calculated by comparison with stenosis measurements on a reference test (CTA or DSA). From the selected studies, the following data were extracted: publication year, population size, whether the study was prospective, which reference test was used, and if there was an indication for selection bias and for verification bias in particular. A formal and systematic review, and meta-analysis, will be performed after the presented new diagnostic study is finished; including its results.

## Results

We identified 6 unique diagnostic series on in-stent stenosis measurements with DUS compared to a reference test (CTA or DSA) [13-18]. In one study DUS and CTA were compared for in-stent measurements, but this paper needed to be excluded because no new criteria were calculated [18]. Additional file 1: Table S1 summarises the 5 series that propose new criteria. In general, the cut-off values are higher than those reported for unstented arteries. For example, the PSV cut-off value for the diagnosis of a >70% stenosis varies between 300 and 450 cm/sec. The reported prevalence of in-stent restenosis was low, which is likely to reflect limited follow-up. Furthermore, the reference test was only performed in these previous studies if stenosis was found on DUS, introducing verification bias. Lal *et al *recently reported a relatively large population of 255 CAS procedures [14]. They confirm overestimation of the degree of in-stent restenosis if regular DUS criteria are applied. Comparisons were made with CTA and DSA. During follow-up, patients underwent DSA only if they were suspected as having restenosis on DUS. Criteria calculated from these data may suffer from verification bias. All patients in their series underwent CTA at the end of their follow-up. The latter data should not suffer from verification bias. In another recent series of Aburahma *et al*, selection was made with DUS and only patients with symptomatic ≥ 50% stenosis or asymptomatic ≥ 80% stenosis were included in their diagnostic study [13]. Applying their DUS criteria to all stented patients is probably not correct because of the selection criteria applied. Zhou *et al *published a large series but concluded that they infrequently found cases of severe stenosis after CAS, and that a multicentre study is warranted to establish reliable in-stent DUS criteria [15]. Only Kwon *et al *reported a series of patients all undergoing both DUS and the reference test, CTA. This study, however, was too small (n = 27) to provide new in-stent cut-off criteria [18].

It is clear from the published data that only small and preselected populations with in-stent restenosis have been studied to date. A large study with sufficient patients with restenosis is therefore needed, as authors of the listed papers also recognised.

### Verification bias

**Figure 2:**
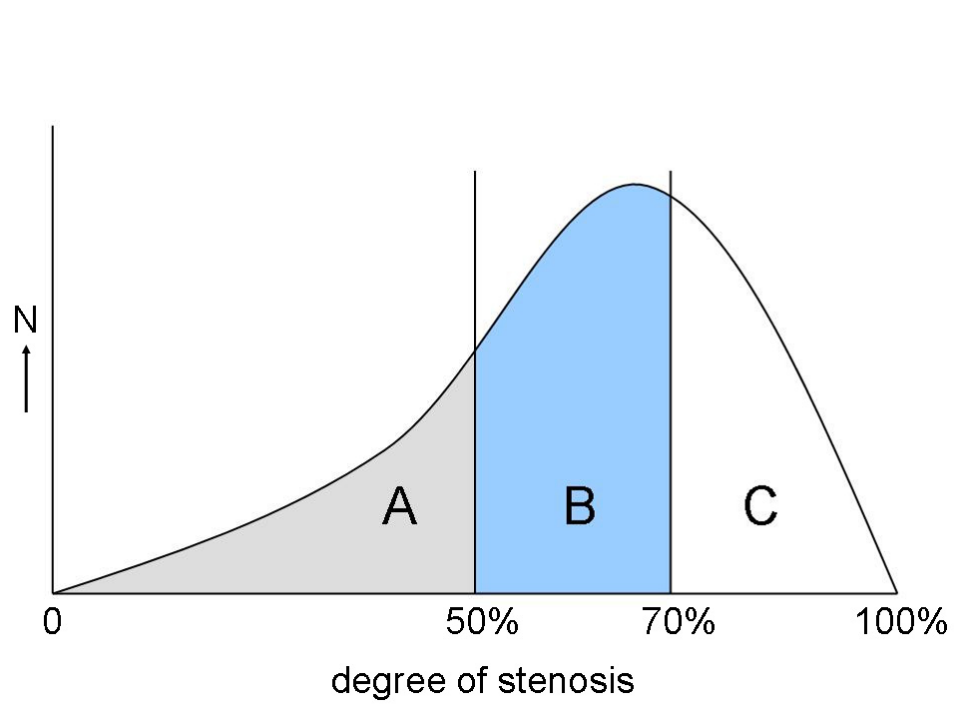
**Example of the effect of verification bias in a hypothetical true distribution of degree restenosis in patients treated with a carotid artery stent**. If a DUS cut-off value of for example 50% stenosis is used to select patients for a diagnostic study, only the patients in part B and C are included and will undergo the reference test. Patients in part A are excluded. The number of patients below a certain threshold, for example 70% is too low (proportion B instead of proportion A and B) and therefore leads to incorrect estimates of the diagnostic value, the specificity in particular. Therefore, DUS criteria obtained from a diagnostic study with comparison with the reference test in part B and C, can not be applied to routine clinical practice for all stented patients (part A, B, and C).

Previous studies often were retrospective, or the reference test (DSA or CTA) was carried out only when a patient was suspected of having restenosis at DUS, which may result in verification bias. Verification bias is introduced if the decision to perform the reference standard procedure depends on the results of the test under investigation, precluding a reliable estimate of the diagnostic accuracy of the latter [19]. The effect of verification bias is explained in figure 2. In a diagnostic study in carotid artery stenosis, in order to obtain valid results, during the follow-up *all *patients need to undergo both the reference test and the test under investigation, regardless of the degree of stenosis on the initial DUS test. If patients were selected based on duplex cut-offs before treatment with CAS, this does not influence the results. On the contrary, the population selected based on age, gender, symptoms, and degree of stenosis prior to treatment, is exactly the domain for a diagnostic study with this purpose.

## Protocol for proposed diagnostic study

To address the deficiencies of the existing studies, we plan to conduct a prospective cohort study nested within the International Carotid Stenting Study (ICSS), an international multi-centre trial in which over 1,700 patients with recently symptomatic internal carotid stenosis have been randomised in equal proportions between stenting and endarterectomy. The ICSS completed recruitment at the end of 2008. The trial protocol specifies annual clinical and ultrasound follow up. A sufficiently large population can be enrolled and because the study is embedded in the ICSS, long term follow-up is guaranteed, allowing us to obtain a larger number of restenoses and therefore more precise estimates for DUS cut-off criteria. Most importantly, during the follow-up all patients in the sub-study will undergo both the reference test of CTA and the test under investigation, regardless of the degree of stenosis on the initial DUS test.

### Reference test

In the protocol, we will use CTA as the reference test for in-stent stenosis measurements. A diagnostic test that provides clear images of the lumen of the internal carotid artery is crucial, because a NASCET-like stenosis measurement is necessary as a reference to estimate the optimal PSV cut-offs for DUS. DSA would be preferable for optimal images of the lumen of stented (carotid) arteries. However, in our opinion, this test would not be ethical anymore as reference test in this diagnostic study because of the small but not negligible complication rate. CTA and MRA are non- or minimally invasive tests providing good lumen images, enabling a 'NASCET'-like stenosis measurement. MRA is not suitable for visualisation of the lumen in a stent, due to artefacts in the magnetic field caused by the material of the stent. Therefore, CTA is the better choice. The use of intravenous iodinated contrast in CTA allows excellent images of the lumen of the arteries. To date, CTA offers high spatial resolution and contrast resolution, and it is a fast technique. Currently, the quality of imaging of the lumen of the artery with CTA is comparable to DSA. We realise that CTA is better validated for non-stented than for stented (carotid) arteries. The diagnostic accuracy of CTA compared to DSA, to diagnose a 70–99% stenosis, was calculated in several studies [8,10]. Wardlaw et al., in a recent meta-analysis, reported a sensitivity and specificity of 77% (95% CI: 68–84%) and 95% (95%CI: 91–97%) respectively [8]. Also, CTA may have certain pitfalls in measuring in-stent stenosis like blurring artefacts in the stent. However, given the limitations, we think CTA is the best choice as a reference test in the assessment of stenosis measurements [20].

Crucial is the fact that we can compare DUS results to a technique that provides clear images of the lumen, in order to investigate if the PSV raises more in a stented artery than in an unstented artery when the remaining lumen is the same as. Because patients will be recruited from multiple centres, the CT scanners will not be identical and we can only handle this limitation by using comparable scan protocols and similar post-processing techniques. Also, different types of stents will be used. If these stents do not have the same physical properties, the PSV's are perhaps slightly different as well [21]. We shall collect data about the used stents, and in our analyses we shall investigate if there is a relation between type of stent and blood flow parameters. However, the advantage of a multi-centre study is that the results will be widely applicable.

### Patients

The diagnostic tests will be performed during routine follow-up of the ICSS trial [3]. The inclusion and exclusion criteria are listed in Table 1. In short, all patients with a symptomatic atheromatous carotid stenosis, ≥ 50% by NASCET criteria, suitable for stenting and surgical endarterectomy, can be included for treatment. In addition to the general ICSS criteria, patients are excluded if they have a contraindication for the contrast agent used for the CTA, such as renal failure. In the ICSS, patients have scheduled follow-up visits at 30 days after treatment, 6 months after randomisation and then annually. We will ask all patients who received a stent to participate in this diagnostic study at their follow-up visit 1 year after treatment, or, for patients included more than one year ago, at the first (yearly) ICSS follow-up visit thereafter. A separate informed consent for this diagnostic sub-study informing the patient about the risks of the extra CTA test is obtained. The sub-study has received approval from the Multicentre Research Ethics Committee in the UK. The complete protocol of ICSS and of the present diagnostic sub study is available at: .

**Table 1:** Inclusion criteria International Carotid Stenting Study (ICSS)

Inclusion criteria
- Symptomatic, extracranial, internal or bifurcation, atheromatous carotid artery stenosis that is suitable for both stenting and surgery and is deemed by the randomising clinician to require treatment.
- The severity of the stenosis of the randomised artery should be at least 50% (as measured by NASCET method or non-invasive equivalent).
- Symptoms must have occurred in the 12 months before randomisation. It is recommended that the time between symptoms and randomisation should be less than 6 months, but patients with symptoms occurring between 6 and 12 months may be included if the randomising physician considers treatment indicated.
- The patient must be clinically stable following their most recent symptoms attributable to the stenotic vessel.
- Patients must be willing to have either treatment, be able to provide informed consent, and be willing to participate in follow-up.
- Patients must be able to undergo their allocated treatment as soon as possible after randomisation.
- Any age greater than 40 may be included. There is no upper age limit.
- Patients should only be randomised if the investigator is uncertain which of the two treatments is best for that patient at that time.
Exclusion criteria
- Patients refusing either treatment.
- Patients unable or unwilling to give informed consent.
- Patients unwilling or unable to participate in follow-up for whatever reason.
- Patients who have had a major stroke with no useful recovery of function within the territory of the treatable artery.

### Sample size

In a sample size calculation in a diagnostic study, in addition to an estimation of the prevalence of disease, a precision of the diagnostic accuracy needs to be defined. We estimate a prevalence of 10 to 20% in-stent restenosis (≥50%) one year after treatment with a stent. To obtain estimates of sensitivity of approximately 90% with a confidence interval of maximally 10%, and a prevalence of restenosis after one year of 20%, we would require a minimum of 172 patients [22]. The estimate of 20% re-stenosis is at the upper limit of the values reported in literature. We used this estimate, because our follow-up interval (after 1 year or more) will be later than of most published numbers. If we use 10% restenosis in the sample size calculation, with similar estimates for sensitivity, specificity, and the confidence interval(s), the number exceeds 300 patients (approximately 350). Because the ICSS included more than 800 patients treated with a stent, and because the follow-up of the ICSS will continue for several years, we expect inclusion of a sufficient high number of patients.

### Diagnostic tests

During the DUS examination, different blood flow velocity parameters will be recorded. The peak systolic velocity (PSV) in the stent will be used as our outcome variable because it is considered the most accurate estimator of the degree of stenosis for DUS [11]. CTA is used as the reference test for in-stent stenosis measurements. Because different centres participate, we will work with different CT machines. However, we will match the protocols. Most importantly, for the assessment of the degree of stenosis, multiplanar (MPR) or curved planar reconstructions (CPR) need to be made [20].

### Stenosis measurement

Two independent observers, blinded for clinical information and for the results of the other diagnostic tests, will perform a stenosis measurement on the reconstructed CTA images, following the Standards for Reporting of Diagnostic Accuracy (STARD) guidelines [23]. The grade of stenosis will be measured according to the NASCET criteria [1]. Stenosis measurements in NASCET were done with DSA. The degree of stenosis was defined as the diameter of the remaining lumen at the stenosis as percentage of the normal lumen distal to the stenosis. The projection, lateral, posteroanterior, or oblique, which shows the most severe stenosis, is used for establishing the degree of stenosis. Measurements with CTA should preferable be done in a comparable manner, in order to correctly apply the trial-data to the clinical decisions about CEA. Thus, for a valid comparison with DSA, the percentage of stenosis will be measured on the MPR or CPR post-processed images of the internal carotid artery, using the three projections mentioned above only.

### Data analysis

Receiver operating characteristics (ROC) curves will be constructed for the diagnoses of 70%–99% and 50%–69% stenosis. The associated optimal sensitivities, specificities, and peak-systolic-velocity thresholds will be derived from the ROC curves. The main result of the analyses will be optimal cut-off points of duplex for in-stent restenosis (not the diagnostic accuracy of duplex) within the context of the present study.

### Meta-analysis

When the present study is completed, we will pool our data with the other diagnostic studies on this particular topic available in literature [13-18]. In addition to pooled weighted estimates of the overall diagnostic accuracy, the data will be modelled using summary ROC analyses. With this model it will be possible to investigate the effect of important quality criteria of the individual studies on the diagnostic accuracy, such as sample size, duration of follow-up, and presence of verification bias.

## Discussion

Carotid angioplasty with stenting (CAS) is being evaluated in ongoing randomized trials. An important factor in the evaluation of carotid artery stents is the degree of possible in-stent restenosis. DUS is a fast and easy test to asses the degree of stenosis. Whereas this diagnostic tool is well validated for stenosis measurements in un-stented carotid arteries, precise cut-off criteria for stented carotid arteries are not available yet. The aim of the proposed study is to validate the use of DUS for in-stent stenosis measurements during follow-up after CAS and to determine reliable cut-off criteria for the different degrees of stenosis. Valid criteria are needed for future research and clinical decision making in patients treated with a carotid artery stent. In conclusion, the ongoing ICSS study provides a unique opportunity to obtain valid DUS in-stent criteria in a sufficiently large population, with a long follow-up and expected high prevalence of restenosis. Verification bias will be avoided by performing both DUS and CTA in all patients. Afterwards, our data will be pooled with other studies, taking into account the limitations of individual studies.

## Abbreviations

CAS: carotid angioplasty with stenting; CEA: carotid endarterectomy; CTA: CT angiography; DUS: duplex ultrasound; PSV: peak systolic velocity; ICSS: International Carotid Stenting Study.

## Competing interests

The authors declare that they have no competing interests.

## Authors' contributions

PJN has designed the sub study and wrote the manuscript. MMB has corrected and approved the study protocols and all versions of the current paper. Both authors read and approved the final manuscript.

## Authors' information

PJN is Neurologist and Clinical Epidemiologist and did previous research in the field of carotid artery stensosis; with special interest in diagnostic study design. MMB is Professor of Stroke Medicine and Consultant Neurologist and has a large track record in stroke research and previous randomised trials; currently he is principal investigator of the International Carotid Stenting Study.

## Pre-publication history

The pre-publication history for this paper can be accessed here:



## Supplementary Material

Additional file 1 **Table S1**. previously reported DUS cut-off values for stenosis measurements within a stent. Click here for file

## References

[B1] Rothwell PM, Eliasziw M, Gutnikov SA, Fox AJ, Taylor DW, Mayberg MR, Warlow CP, Barnett HJ, Carotid Endarterectomy Trialists' Collaboration. Carotid Endarterectomy Trialists' Collaboration (2003). Analysis of pooled data from the randomised controlled trials of endarterectomy for symptomatic carotid stenosis. Lancet.

[B2] Hobson RW (2002). Update on the Carotid Revascularization Endarterectomy vs. Stent Trial (CREST) protocol. J Am Coll Surg.

[B3] Featherstone RL, Brown MM, Coward LJ, ICSS Investigators (2004). International carotid stenting study: protocol for a randomised clinical trial comparing carotid stenting with endarterectomy in symptomatic carotid artery stenosis. Cerebrovasc Dis.

[B4] Brown MM, Hacke W (2004). Carotid artery stenting: the need for randomised trials. Cerebrovasc Dis.

[B5] Brown MM (2004). Should carotid stenting replace carotid endarterectomy in routine clinical practice?. Cerebrovasc Dis.

[B6] McCabe DJ, Pereira AC, Clifton A, Bland JM, Brown MM, CAVATAS Investigators (2005). Restenosis after carotid angioplasty, stenting, or endarterectomy in the Carotid and Vertebral Artery Transluminal Angioplasty Study (CAVATAS). Stroke.

[B7] Eckstein HH, Ringleb P, Allenberg JR, Berger J, Fraedrich G, Hacke W, Hennerici M, Stingele R, Fiehler J, Zeumer H, Jansen O (2008). Results of the Stent-Protected Angioplasty versus Carotid Endarterectomy (SPACE) study to treat symptomatic stenoses at 2 years: a multinational, prospective, randomised trial. Lancet Neurol.

[B8] Wardlaw JM, Chappell FM, Best JJ, Wartolowska K, Berry E, NHS Research and Development Health Technology Assessment Carotid Stenosis Imaging Group (2006). Non-invasive imaging compared with intra-arterial angiography in the diagnosis of symptomatic carotid stenosis: a meta-analysis. Lancet.

[B9] Nederkoorn PJ, Graaf Y van der, Hunink MG (2003). Duplex ultrasound and magnetic resonance angiography compared with digital subtraction angiography in carotid artery stenosis: a systematic review. Stroke.

[B10] Koelemay MJ, Nederkoorn PJ, Reitsma JB, Majoie CB (2004). Systematic review of computed tomographic angiography for assessment of carotid artery disease. Stroke.

[B11] Hunink MG, Polak JF, Barlan MM, O'Leary DH (1993). Detection and quantification of carotid artery stenosis: efficacy of various doppler velocity parameters. AJR Am J Roentgenol.

[B12] Lal BK, Hobson RW, Goldstein J, Chakhtoura EY, Durán WN (2004). Carotid artery stenting: is there a need to revise ultrasound velocity criteria?. J Vasc Surg.

[B13] Aburahma AF, Abu-Halimah S, Bensenhaver J, Dean LS, Keiffer T, Emmett M, Flaherty S (2008). Optimal carotid duplex velocity criteria for defining the severity of carotid in-stent restenosis. J Vasc Surg.

[B14] Lal BK, Hobson RW, Tofighi B, Kapadia I, Cuadra S, Jamil Z (2008). Duplex ultrasound velocity criteria for the stented carotid artery. J Vasc Surg.

[B15] Zhou W, Felkai DD, Evans M, McCoy SA, Lin PH, Kougias P, El-Sayed HF, Lumsden AB (2008). Ultrasound criteria for severe in-stent restenosis following carotid artery stenting. J Vasc Surg.

[B16] Chi YW, White CJ, Woods TC, Goldman CK (2007). Ultrasound velocity criteria for carotid in-stent restenosis. Catheter Cardiovasc Interv.

[B17] Stanziale SF, Wholey MH, Boules TN, Selzer F, Makaroun MS (2005). Determining in-stent stenosis of carotid arteries by duplex ultrasound criteria. J Endovasc Ther.

[B18] Kwon BJ, Jung C, Sheen SH, Cho JH, Han MH (2007). CT angiography of stented carotid arteries: comparison with Doppler ultrasonography. J Endovasc Ther.

[B19] Begg CB, Greenes RA (1983). Assessment of diagnostic tests when disease verification is subject to selection bias. Biometrics.

[B20] Nederkoorn PJ, Majoie CB, Stam J, Gillard J (2007). Computed tomographic angiography of carotid artery stenosis. Carotid diseases, the role of imaging in diagnosis and management.

[B21] Spies C, Doshi R, Spoon J, Snell RJ (2007). Carotid artery stent type influences duplex ultrasonography derived peak systolic velocity: findings of an in-vitro model. Catheter Cardiovasc Interv.

[B22] Flahault A, Cadilhac M, Thomas G (2005). Sample size calculation should be performed for design accuracy in diagnostic test studies. J Clin Epidemiol.

[B23] Bossuyt PM (2003). Towards complete and accurate reporting of studies of diagnostic accuracy: The STARD. Ann Intern Med.

