# Supplementary material for: Optimal cut-off criteria for duplex ultrasound for the diagnosis of restenosis in stented carotid arteries: Review and protocol for a diagnostic study
Source: BMC Neurol. 2009 Jul 22;9:36. doi: 10.1186/1471-2377-9-36 (PMC2722571; doi:10.1186/1471-2377-9-36)
Supplement: Additional file 1 — Table S1. previously reported DUS cut-off values for stenosis measurements within a stent. [file 1471-2377-9-36-S1.doc]

**Table 1.** Previously reported DUS cut-off values for stenosis measurements within a stent.

| First Author | Publication year | Population  Size (N) | Prospective | Subgroup selection* | Subgroup (N) | Reference test | PSV**  ≥20% | PSV  ≥30% | PSV  ≥50% | PSV ≥70% | PSV ≥80% |
| --- | --- | --- | --- | --- | --- | --- | --- | --- | --- | --- | --- |
| Aburhama^13^ | 2008 | 144 | Yes | Yes | 144 | CTA |  | 178 | 278 |  | 403 |
| Lal^14^ | 2008 | 225 | No | Yes | 99 | CTA or DSA | 150 |  | 220 |  | 340 |
| Zhou^15^ | 2008 | 256 | No | Yes | 22 | DSA |  |  |  | 300 |  |
| Chi^16^ | 2007 | 260 | Yes | Yes | 13 | DSA |  |  | 240 | 450 |  |
| Stanziale^17^ | 2005 | 605 | No | Yes | 118 | DSA |  |  | 225 | 350 |  |

* Selection of a subgroup based on DUS results (the test under evaluation) indicates possible verification bias. These subgroups are compared to the reference test in order to obtain the listed DUS cut-off values.

** Suggested cut-offs for in-stent restenosis measurements based on the peak systolic velocity (PSV); in cm/sec.
